# Supplementary material for: Brain-wide neural recordings in mice navigating physical spaces enabled by a cranial exoskeleton
Source: Res Sq. 2023 Nov 13:rs.3.rs-3491330. Preprint. [Version 1] doi: 10.21203/rs.3.rs-3491330/v1 (PMC10680923; doi:10.21203/rs.3.rs-3491330/v1)
Supplement: Supplement 1 [file NIHPPrs3491330v1-supplement-1.pdf]

LIST OF SUPPLEMENTARY MATERIALS

1) Extended data figures: Document consisting of nine captioned figures containing data and schematics that support the results and methods presented in the main text.

2) Supplementary Info: Document consisting of eight captioned figures containing data and schematics that support the results and methods presented in the main text.

3) Supplementary software and Supplementary CAD files: Can be found at GitHub Repository: https://github.com/bsbrl/exoskeleton

4) Supplementary Videos: Five captioned videos containing video data and schematics that support the results and methods presented in the main text.

Supplementary Video 1: Velocity-acceleration profiles of freely behaving mice and mice maneuvering the exoskeleton during tuning of the x axis.

Video (8x speed) showing top-down view of a mouse in open field arena with several body points labelled using labelled using DeepLabCut markerless tracking software (left window), and the corresponding velocity-acceleration profile for forwards/backwards motion in the mouse's frame of reference (grey; right window). Transition at half-way to a mouse maneuvering the exoskeleton around the linear oval track with tuned mass and damping values and the corresponding velocity-acceleration profile (blue).

Supplementary Video 2: Gait dynamics of freely behaving mice and mice maneuvering the exoskeleton.

Video (1x speed) showing a side-on-vide of a freely behaving mouse locomoting along a 27 cm long straight section of the linear oval track (left window), with left paws and other body points labelled using DeepLabCut marker-less tracking software, and the corresponding gait metrics for steps in the video (large grey dots) overlaid on top of the gait metrics for all mice (right window). Transition at half-way to the same mouse maneuvering the exoskeleton with metrics for steps in the video (large blue dots) overlaid on top of the gait metrics for all mice.

Supplementary Video 3: Mouse performing the navigational decision-making task while maneuvering the exoskeleton.

Video (2x speed) showing a trained mouse performing the navigational decision-making task (sensory-cue guided alternating choice) while maneuvering the exoskeleton. Top half of the video shows (left) view from behavioral camera mounted to the headstage and (top-right and bottom-right) views from 2 cameras placed around the behavioral arena. The bottom half of the video shows (top panels) the force time series in x, y and yaw, and (bottom panels) the velocity and acceleration profiles in x, y and yaw, where thick lines indicate the mouse is in the turning zone.

Supplementary Video 4: Mescoscale cellular resolution imaging of the dorsal cortex of a mouse navigating the 8-maze arena

Video (8x speed) showing a mouse completing 16 turns in the 8-maze arena while maneuvering the imaging headstage on the exoskeleton (top-left window), with corresponding video of fluorescence in the mouse's cortex captured using the imaging headstage (bottom-left window).

Single cell activity is visible in the four, 1x1 mm, regions of interest (ROIs) (right windows). Transition half-way to a maximum intensity image of the brain (bottom-left window), and to plot of the ΔF/F traces from all cells in each ROI (right windows).

Supplementary Video 5: Multi-site electrophysiology recordings in a mouse performing the navigational decision-making task in the 8-maze arena

Video (10x speed) showing a mouse on the disk treadmill (top-left window) as probes are inserted through the cranial implant (bottom-left window) into the two anterior probe insertion sites (top-right window). Transition at half-way to video (2x speed) of the mouse completing a correct right turn decision (top-left window) with a corresponding plot of voltage signal traces from 23 channels across the 2 neural probes showing spiking signals, where colored bar indicates the probe (bottom left window; dark blue, M1; light blue, M1/SS). In the right window, the mean waveforms of 24 cells identified on the 2 probes are shown (thick colored lines; dark blue, M1; light blue, M1/SS) overlaid with the spiking activity of these cells (thin black line) as the spikes occur.

Supplementary Information

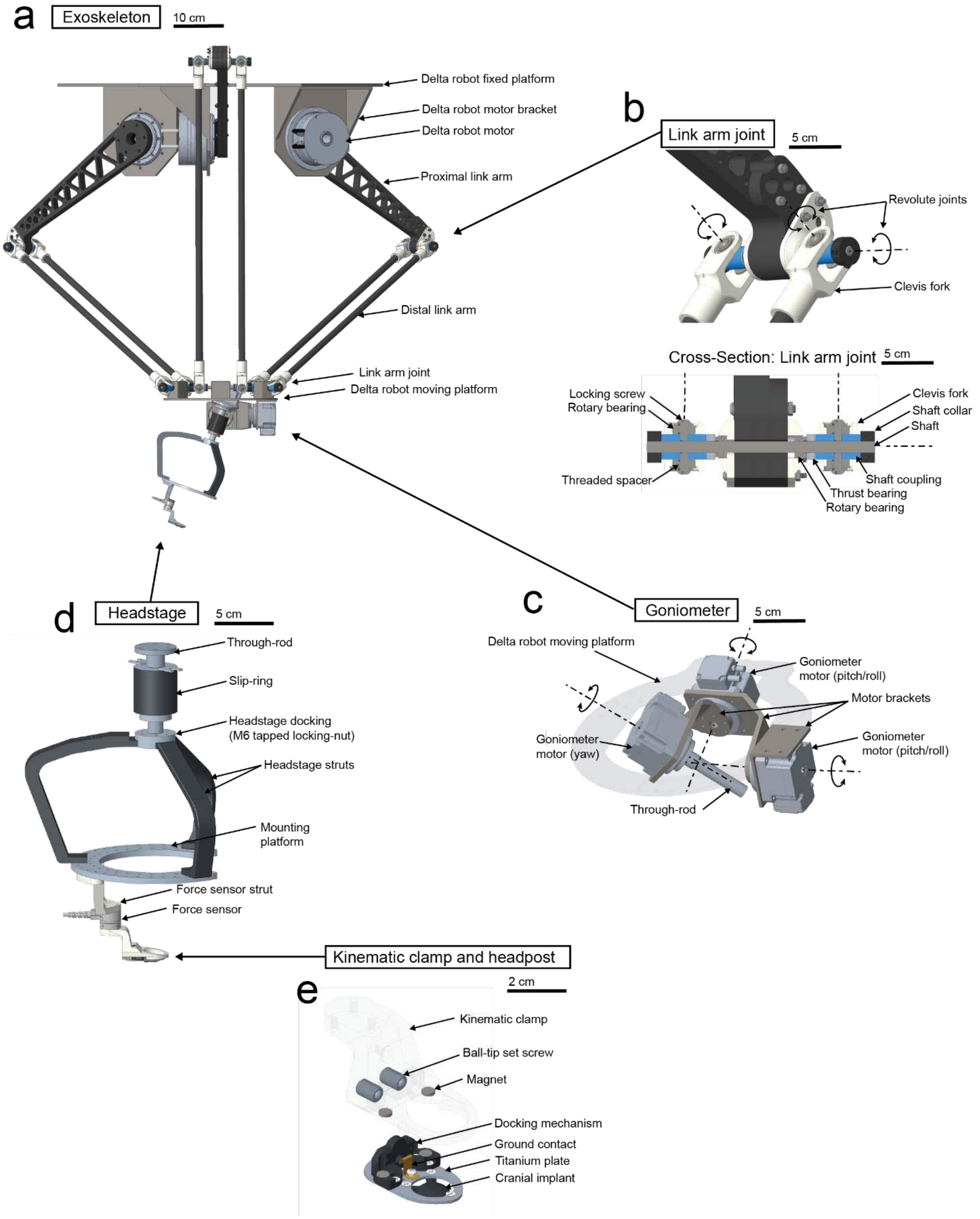

Supplementary Information 1: Exoskeleton construction

(a) Exoskeleton delta robot, where each of the 3 motors are connected to the moving platform via a proximal arm, a pair of distal link arms, and a pair of link arm joints.
(b) Delta robot link arm joint (top) and cross-section through a pair of orthogonal revolute joints showing the bearing component-stack (bottom).
(c) Goniometer mounted within the moving platform of the delta robot, where 3 motors configured orthogonal to one another generate pitch/roll/yaw motion.
(d) Headstage without any neural or behavioral monitoring equipment mounted to it, with the slip-ring at the top and the force sensor at the bottom.
(e) Kinematic clamp and chronically implanted headpost, where ball-tip set screws and magnets facilitate docking of the mouse to the headstage.

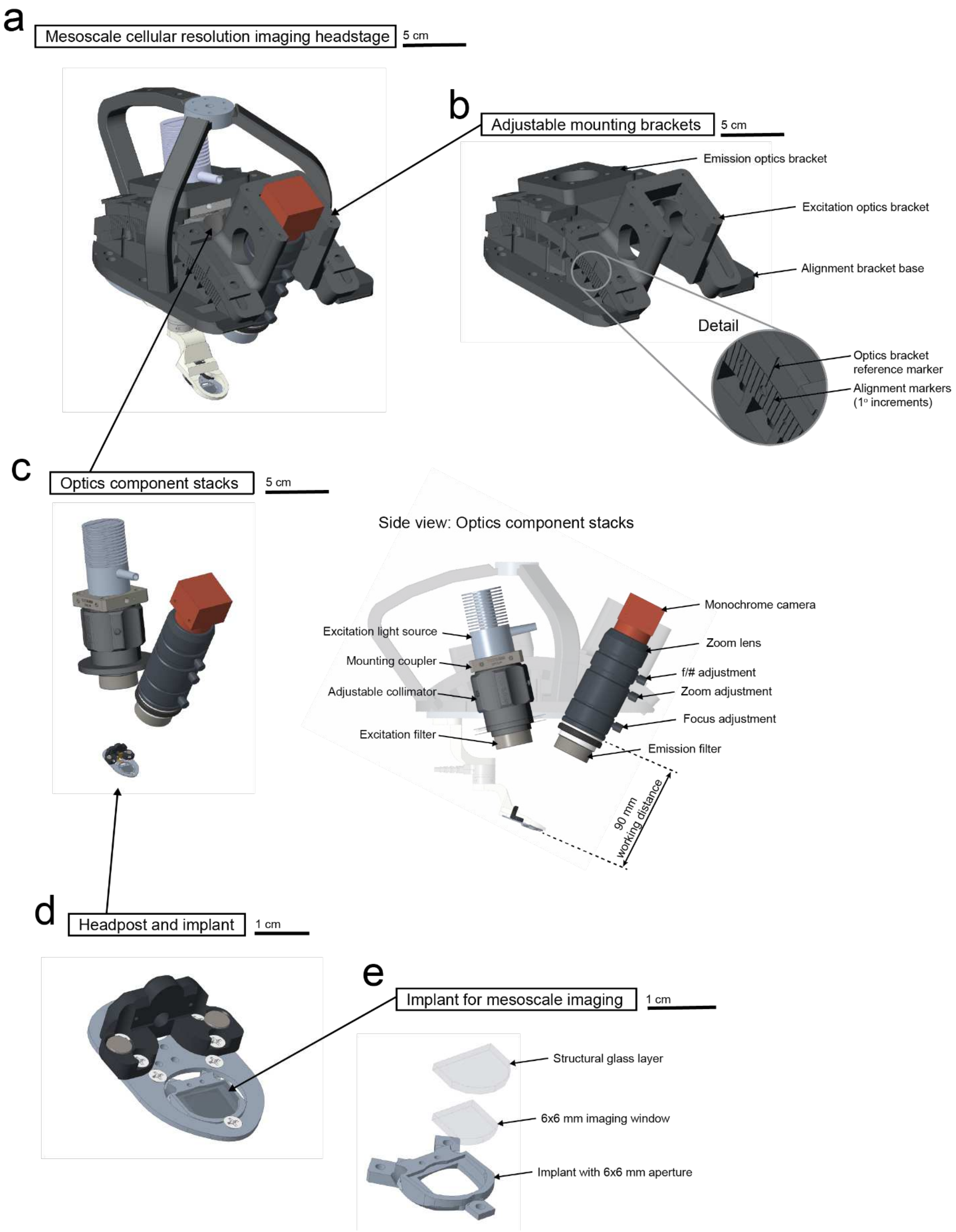

a
Mesoscale cellular resolution imaging headstage
5 cm
b
Adjustable mounting brackets
5 cm
Emission optics bracket
Excitation optics bracket
Alignment bracket base
Detail
Optics bracket reference marker
Alignment markers (1° increments)
c
Optics component stacks
5 cm
Side view: Optics component stacks
Monochrome camera
Zoom lens
f/# adjustment
Zoom adjustment
Focus adjustment
Emission filter
Excitation light source
Mounting coupler
Adjustable collimator
Excitation filter
90 mm working distance
d
Headpost and implant
1 cm
e
Implant for mesoscale imaging
1 cm
Structural glass layer
6x6 mm imaging window
Implant with 6x6 mm aperture

Supplementary Information 2: Headstage configuration for mesoscale, cellular resolution imaging

(a) Headstage configuration for mesoscale imaging using 1-photon excitation.
(b) Mounting brackets for the emission and excitation optics stacks can have their angle adjusted with respect to the imaging plane on the mouse’s implant.
(c) Component stacks (left) for the emission and excitation optics and (right) side-on-view of these component stacks within the headstage with components and working distance annotated.
(d) Headpost and implant for mesoscale imaging, that are chronically attached to the mouse.
(e) Exploded view of the implant for mesoscale imaging, which is surgically implanted over the mouse’s brain, showing the 2 layers of the glass.

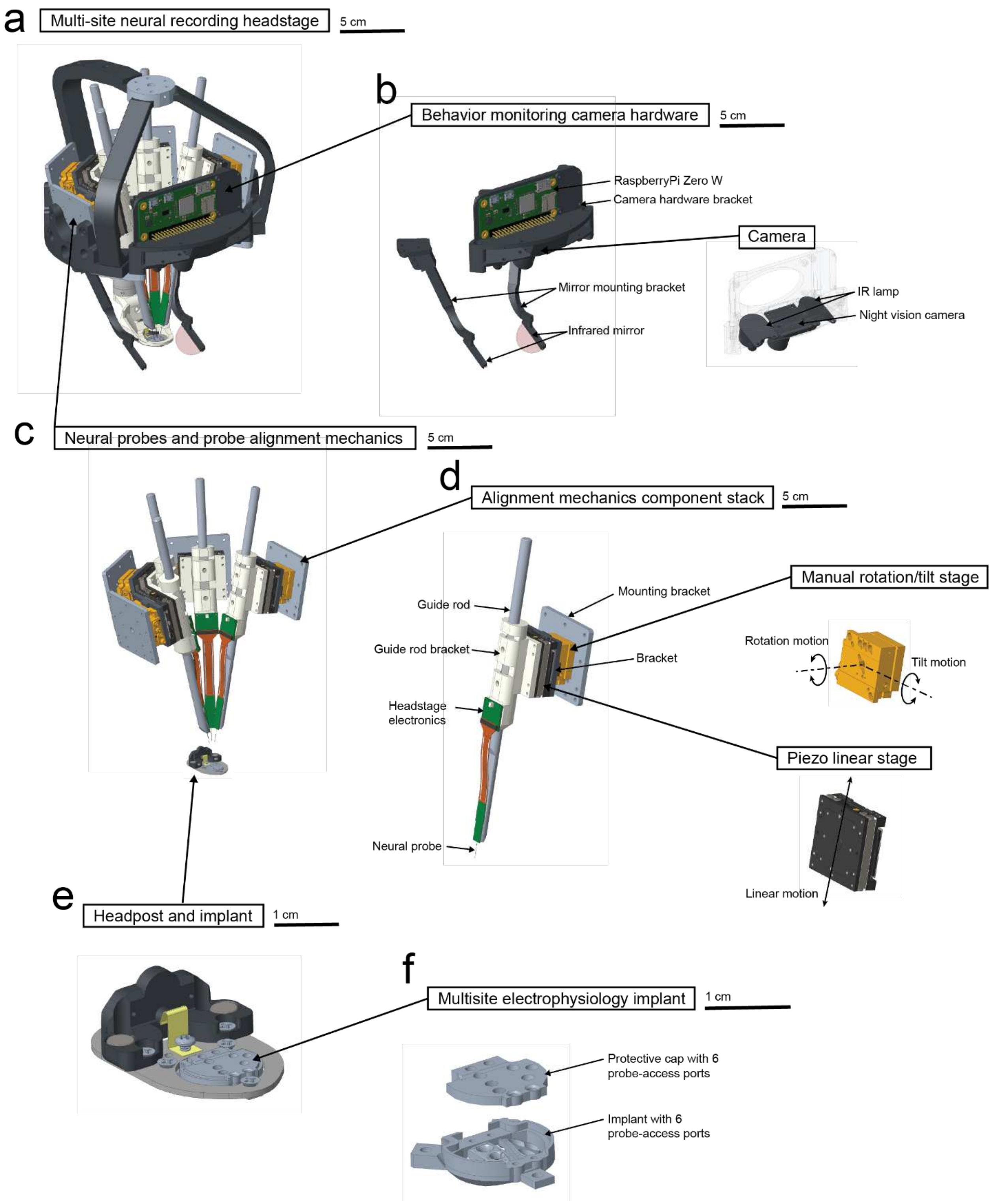

a
Multi-site neural recording headstage
5 cm
b
Behavior monitoring camera hardware
5 cm
RaspberryPi Zero W
Camera hardware bracket
Camera
Mirror mounting bracket
Infrared mirror
IR lamp
Night vision camera
c
Neural probes and probe alignment mechanics
5 cm
d
Alignment mechanics component stack
5 cm
Mounting bracket
Manual rotation/tilt stage
Guide rod
Guide rod bracket
Bracket
Rotation motion
Tilt motion
Headstage electronics
Piezo linear stage
Neural probe
Linear motion
e
Headpost and implant
1 cm
f
Multisite electrophysiology implant
1 cm
Protective cap with 6 probe-access ports
Implant with 6 probe-access ports

Supplementary Information 3: Headstage construction for multi-site electrophysiology and behavioral monitoring

(a) Headstage configuration for multi-site electrophysiology and behavioral monitoring, with 4 Neuropixels probes installed.
(b) Behavioral monitoring camera hardware includes (left) a microcontroller to capture and store video data, optional infrared mirrors to monitor pupil and whisker activity, and (right) a night vision camera with infrared lamps to illuminate the field of view.
(c) Four sets of neural probes and probe alignment mechanics arranged above the headpost.
(d) One set of alignment mechanics and neural probe with components labelled, and the axes of travel of the (top-right) manual rotation/tilt stage used to align the neural probe and the (bottom-right) piezo linear stage used to insert and remove the neural probe.
(e) Headpost and implant for multi-site electrophysiology that are chronically attached to the mouse (for surgical procedure, see **Supplementary Info. 8**).
(f) Exploded view of the implant for multi-site electrophysiology, where silicon is inserted between the implant and the protective cap to protect the exposed brain within the probe access-ports.

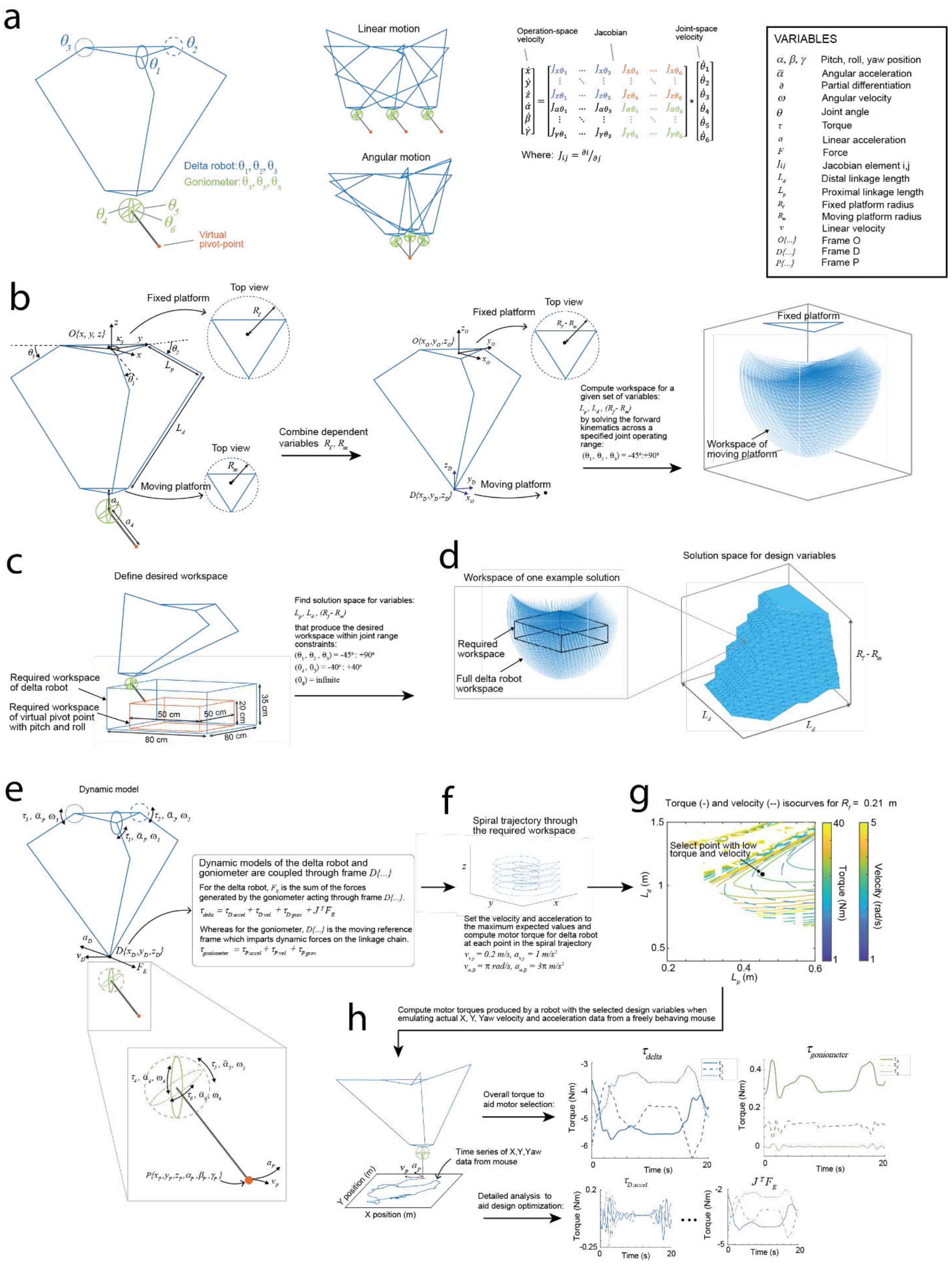
a
Delta robot: θ1, θ2, θ3
Goniometer: θ4, θ5, θ6
Virtual pivot-point
Linear motion
Angular motion
Operation-space velocity
Jacobian
Joint-space velocity
Where: Jij = ∂i/∂j
VARIABLES
α, β, γ Pitch, roll, yaw position
Angular acceleration
Partial differentiation
Angular velocity
Joint angle
Torque
Linear acceleration
Force
Jacobian element i,j
Distal linkage length
Proximal linkage length
Fixed platform radius
Moving platform radius
Linear velocity
Frame O
Frame D
Frame P
b
Fixed platform
Top view
Moving platform
Combine dependent variables Rf, Rm
Compute workspace for a given set of variables: Lp, Ld, (Rf - Rm) by solving the forward kinematics across a specified joint operating range: (θ1, θ2, θ3) = -45°:+90°
Workspace of moving platform
c
Define desired workspace
Required workspace of delta robot
Required workspace of virtual pivot point with pitch and roll
50 cm
50 cm
20 cm
35 cm
80 cm
80 cm
Find solution space for variables: Lp, Ld, (Rf - Rm) that produce the desired workspace within joint range constraints: (θ1, θ2, θ3) = -45°: +90° (θ4, θ5) = -40°: +40° (θ6) = infinite
d
Workspace of one example solution
Required workspace
Full delta robot workspace
Solution space for design variables
e
Dynamic model
Dynamic models of the delta robot and goniometer are coupled through frame D{...}
For the delta robot, FE is the sum of the forces generated by the goniometer acting through frame D{...}.
Whereas for the goniometer, D{...} is the moving reference frame which imparts dynamic forces on the linkage chain.
f
Spiral trajectory through the required workspace
Set the velocity and acceleration to the maximum expected values and compute motor torque for delta robot at each point in the spiral trajectory
g
Torque (-) and velocity (--) isocurves for Rf = 0.21 m
Select point with low torque and velocity
Torque (Nm)
Velocity (rad/s)
h
Compute motor torques produced by a robot with the selected design variables when emulating actual X, Y, Yaw velocity and acceleration data from a freely behaving mouse
Time series of X,Y,Yaw data from mouse
Y position (m)
X position (m)
Overall torque to aid motor selection:
Detailed analysis to aid design optimization:
Torque (Nm)
Time (s)

Supplementary Information 4: Exoskeleton design optimization

(a) Schematics showing the 3 actuated joints in the delta robot and the 3 in the goniometer and motion sequences showing the synchronized movement of these joints during linear and angular motion of the pivot point. The system Jacobian with colors indicating the elements associated with linear (blue), angular (green), and pivoting (orange) motion.
(b) The main design variables for the delta robot (left), combining the fixed and moving platform radii into one variable (center), and delta robot workspace (right).
(c) The desired workspace (range of motion) for the delta robot and its relationship to the desired workspace for the pivot point.
(d) The solution space (combinations of dimensions which can achieve the desired range of motion in the delta robot), with a schematic of one example solution (left).
(e) The main variables in the dynamic model of the exoskeleton.
(f) The spiral trajectory through the workspace and velocity and acceleration parameters set at each point along the trajectory.
(g) The maximum motor torque and joint velocity computed along the spiral trajectory for each of the combinations of dimensions in the solution space in (d), and a point selected with both low torque and low joint velocity.
(h) Time series data acquired from markerless tracking of a freely behaving mouse (left) and the corresponding time series of motor torque acceleration, velocity, gravity, and external force components (right).

a Transfer function model of delta robot and controller in state space

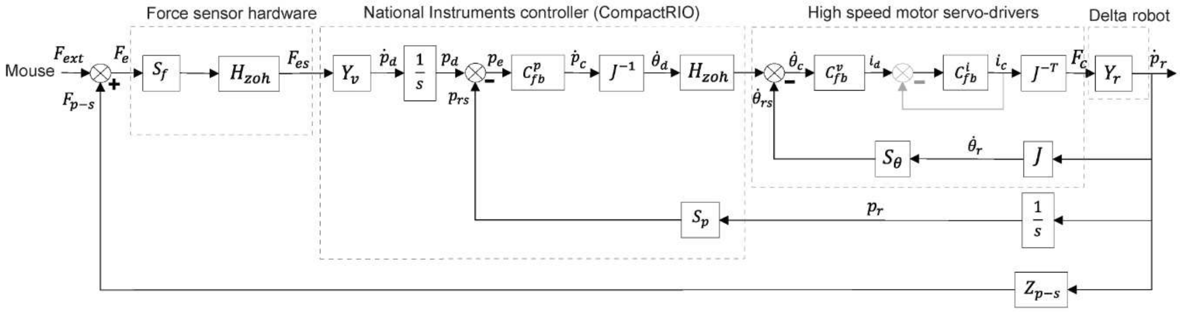

b Node graph of delta robot and controller

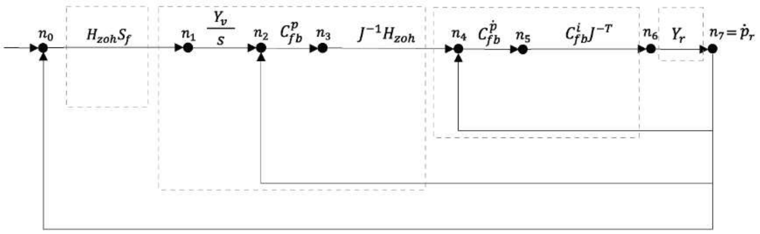

$n_0 = F_{ext} + Z_{p-s}x_7$ $\quad$ $n_1 = H_{zoh}S_f n_0$

$n_2 = \frac{Y_v}{s}n_1 - \frac{S_p}{s}n_7$ $\quad$ $n_3 = C_{fb}^p\ n_2$

$n_4 = J^{-1}H_{zoh}n_3 - S_\theta J n_7$ $\quad$ $n_5 = C_{fb}^{\dot{p}}\ n_4$

$n_6 = J^{-T}C_{fb}^i\, n_5$ $\quad$ $n_7 = \dot{p}_r = Y_r n_6$

Assume $S_\theta = 1,\ C_{fb}^i = 1$

Apparent admittance $Y_a = \dot{p}_r/F_{ext}$

$$\dot{p}_r/F_{ext} = \left(ABC4H_{zoh}S_f\right)/\left(\left(1 + ABCZ_{p-s} + ABD + AJ\right)\right)$$

where:

$A = Y_r J^{-T} C_{fb}^i C_{fb}^{\dot{p}}$ $\quad$ $B = J^{-1}H_{zoh}C_{fb}^p$

$C = \frac{Y_v}{s}$ $\quad$ $D = \frac{S_p}{s}$

VARIABLES

- F Force
- i Current
- J Delta robot Jacobian
- p Position (and velocity $\dot{p}$) in operational space
- S Sensor dynamics (for ideal sensor *S=1*)
- s Laplace variable
- v Velocity (operational space)
- Z Impedance (inverse of admittance)
- θ Position (and velocity $\dot{\theta}$) in joint space
- τ Time constant for first order dynamic system

VARIABLES

$$C_{fb} = k_P + \frac{k_I}{s} + \frac{k_D s}{\tau_I s + 1}$$ PID feedback controller with gains $k_{P,I,D}$

$$H_{zoh} = \frac{1 - e^{-sT_s}}{sT_s}$$ Zero order hold for delay time T

$$Y = \frac{1}{ms + b}$$ Mechanical admittance for mass m damper b system

SUB/SUPERSCRIPTS

- c Commanded
- d Desired
- e Error
- f Force
- ext External
- i Current
- p Position (and velocity $\dot{p}$) in operational space
- $p-s$ Post-sensor
- s Discrete sample of continuous signal
- r Robot
- T Transpose
- v Virtual
- θ Position (and velocity $\dot{\theta}$) in joint space

c Bode plot analysis of delta robot and controller bandwidth

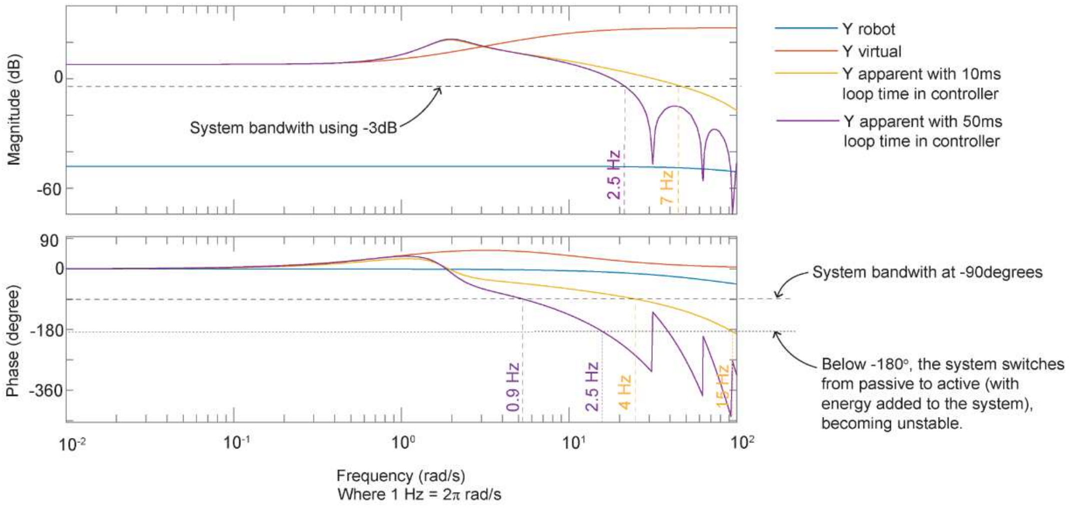

Supplementary Information 5: Evaluation of the system bandwidth using a Laplace space model

(a) A transfer function model of the system (robotic exoskeleton and controller), with variables transformed to Laplace-space.

(b) Node graph of the transfer function model in (a).

(c) Bode plot (top, magnitude vs. frequency; bottom, phase vs. frequency) of the system showing the apparent admittance (yellow and purple) emulates the virtual admittance (orange) well at low frequencies but at higher frequencies degrades and approaches the true robot admittance (blue). Reducing the loop time on the controller from 50 ms (yellow) to 10 ms (yellow) increases the bandwidth from 0.9 – 2.5 Hz to 4 – 7 Hz.

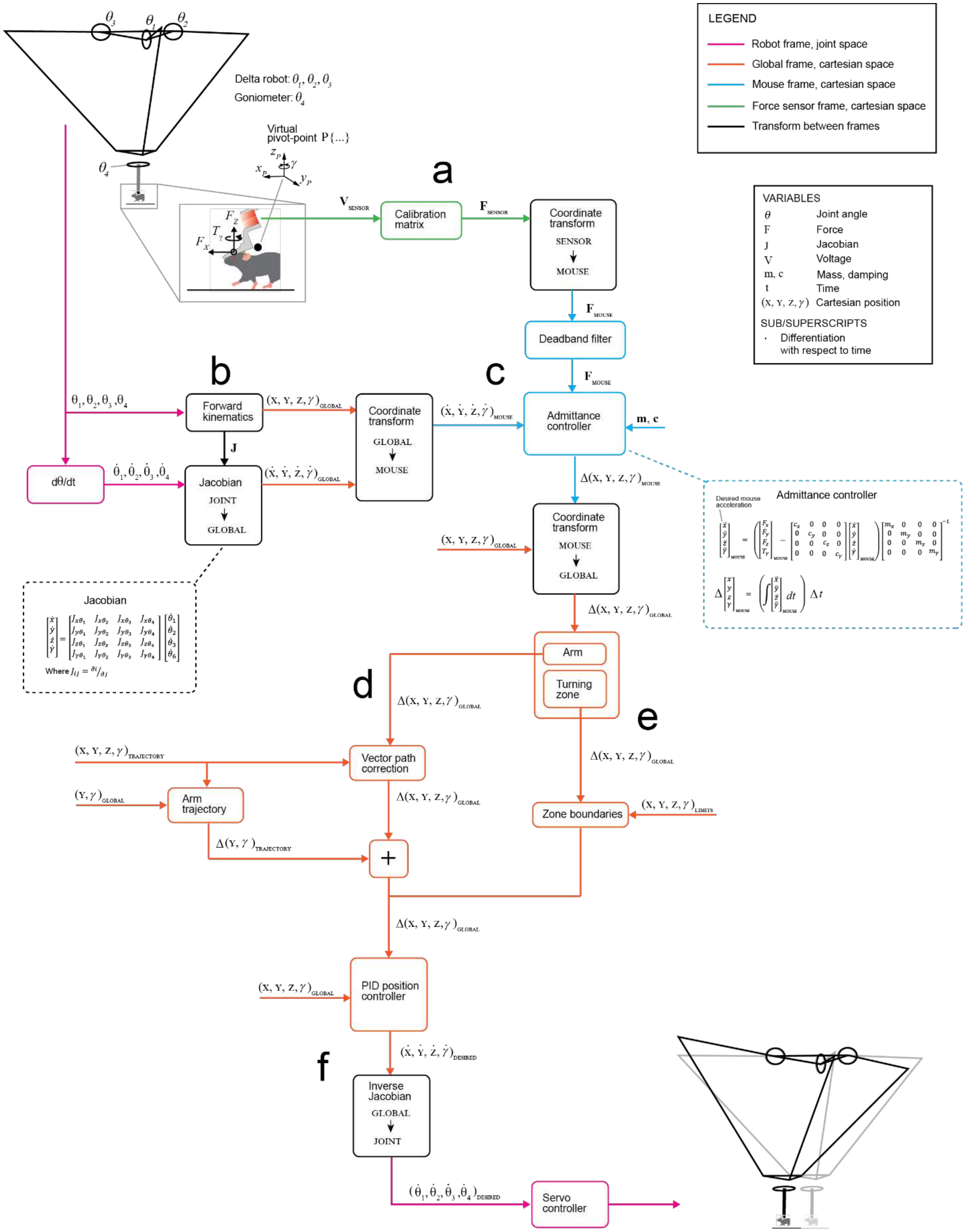

LEGEND
Robot frame, joint space
Global frame, cartesian space
Mouse frame, cartesian space
Force sensor frame, cartesian space
Transform between frames
VARIABLES
θ Joint angle
F Force
J Jacobian
V Voltage
m, c Mass, damping
t Time
(X, Y, Z, γ) Cartesian position
SUB/SUPERSCRIPTS
· Differentiation with respect to time
Delta robot: θ1, θ2, θ3
Goniometer: θ4
Virtual pivot-point P{...}
a
b
c
d
e
f
Calibration matrix
Coordinate transform
SENSOR
MOUSE
Deadband filter
Admittance controller
m, c
Forward kinematics
GLOBAL
dθ/dt
Jacobian
JOINT
Where $J_{ij} = \partial i / \partial j$
Admittance controller
Desired mouse acceleration
Arm
Turning zone
Vector path correction
Arm trajectory
Zone boundaries
PID position controller
Inverse Jacobian
Servo controller

Supplementary Information 6: Flow diagram of the exoskeleton controller in the 8-maze arena

(a) Raw voltages from the force sensor are converted to forces in the force sensor frame using a calibration matrix, and then these forces transformed to the mouse's coordinate frame to describe the mouse's forces.
(b) Forward kinematics converts the joint angles of the robot into the global position of the mouse and to define the Jacobian. The Jacobian converts the joint velocities to global velocities which are then transformed to the mouse's coordinate frame to describe the mouse's velocities.
(c) The mouse's forces and velocities are fed into the admittance controller, along with programmed mass and damping values, to find the desired mouse accelerations. These accelerations are then integrated to find a desired change in position (the product of the desired velocity and controller loop time), which is transformed to the global coordinate frame.
(d) If the mouse is an arm of the 8-maze arena, then the desired change in position is modified using vector path correction and the mouse's Y and Yaw axes are trajectory controlled before being sent to the PID controller (see also **Extended Data 5**).
(e) If the mouse is in the turning zone of the 8-maze arena, then the desired change in position is checked to ensure it is within the zone boundaries before being sent to the PID controller.
(f) The output of the PID controller is desired velocities, which are transformed to joint space using the inverse Jacobian to produce desired joint velocities. These joint velocities are sent to the servo controllers that drive each motor in the robot to produce motion.

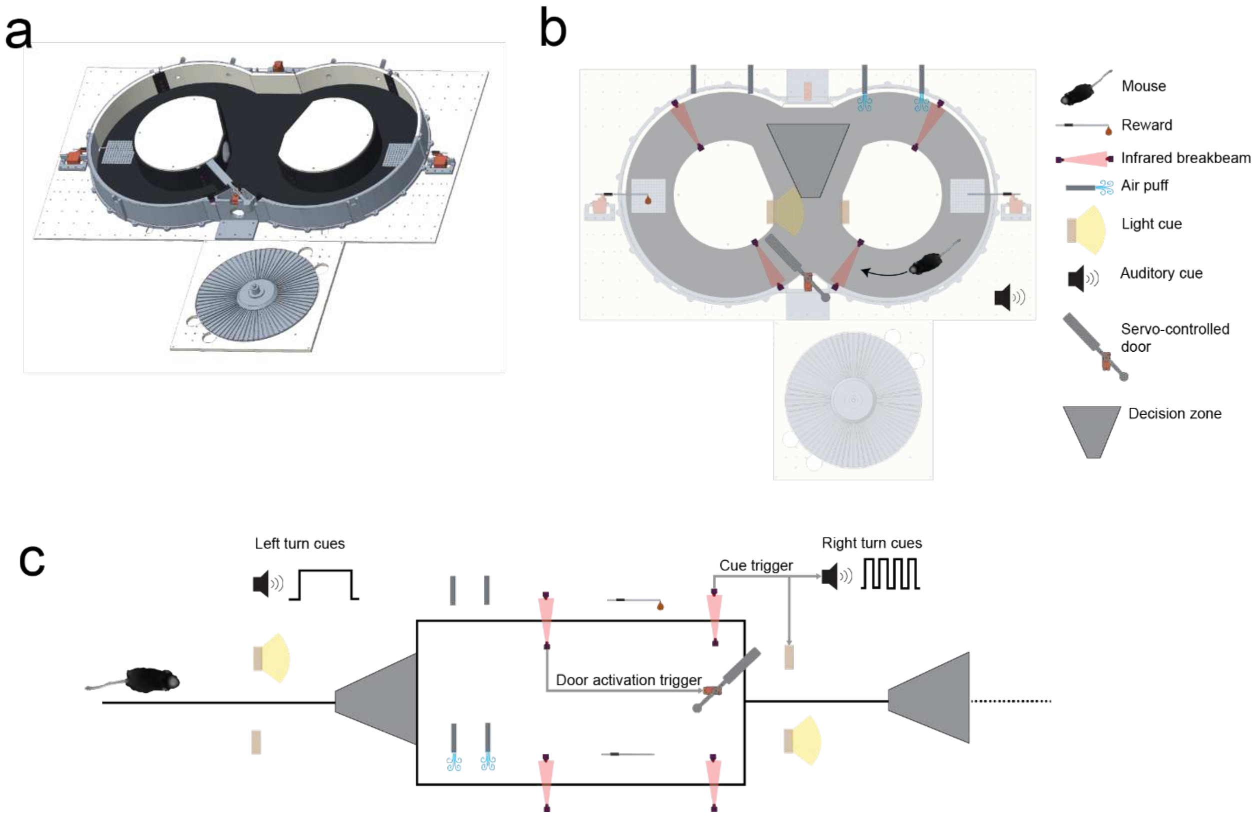

Supplementary Information 7: Navigational decision-making task protocol in the 8-maze arena

(a) CAD rendering of the 8-maze arena.
(b) Top-down view of the 8-maze arena and its elements, with the mouse in the right arm and elements configured for a left turn (right arm air-puff; left arm reward; left turn light cue; servo door turned left).
(c) Linear sequence showing left turn configuration of the 8-maze elements, and the trigger operations of the break-beam sensors.

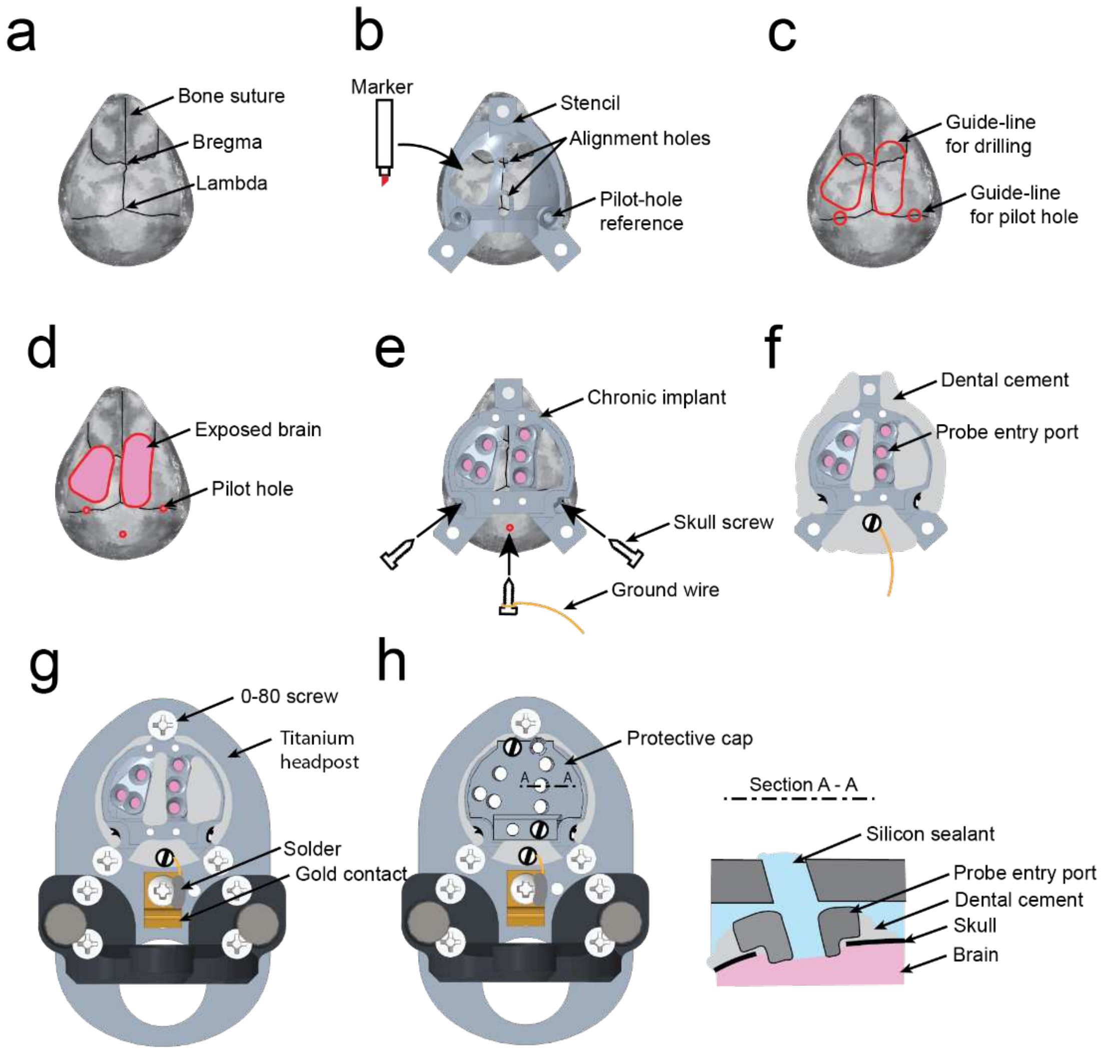

Supplementary Information 8: Surgical procedure for chronical implantation of the headpost and implant for multi-site electrophysiology

(a) Bone sutures and anatomical landmarks visible on the dorsal surface of the skull after removing the scalp and soft connective tissue.
(b) Plastic stencil used to reference the locations of the targeted craniotomies and pilot-holes for skull screws to the locations of the anatomical landmarks on the skull surface.
(c) Dorsal surface of the skull after marking guide-line locations of the targeted craniotomies and pilot-holes for skull screws using the stencil in (b).
(d) Areas of exposed brain tissue after craniotomy and pilot holes for skull screws.
(e) Implant for multi-site electrophysiology is screwed down using 2 skull screws through 2 of the pilot holes, and a third skull screw with a ground wire attached is screwed down through the third pilot hole.
(f) The implant and skull screws are secured using dental cement.
(g) The headpost for electrophysiology is attached to the implant using three 0-80 screws, and the ground wire is soldered onto a gold contact located on the headpost.
(h) A protective cap is screwed on to the implant using self-tapping screws (skull screws) after applying silicon sealant over the exposed brain. Before recording sessions, this silicon sealant is replaced with a silicone-gel.

GitHub repo

CAD

(a) Exoskeleton construction
(b) Electrophysiology headstage construction
(c) Imaging headstage construction
(d) Linear oval track arena
(e) 8-maze arena

Software

(a) LABVIEW files for exoskeleton control on the CompactRIO
(b) LABVIEW files for 8-maze control on the CompactRIO (freely behaving training)

Data analysis

MATLAB files and raw data for:

(a) Exoskeleton design optimization
(b) Exoskeleton admittance controller tuning
(c) Gait analysis
(d) 8-maze task performance analysis
(e) Mesoscale imaging data analysis during 8-maze navigation
(f) Electrophysiology data analysis during 8-maze alternating choice task

Supplementary Files

This is a list of supplementary files associated with this preprint. Click to download.

- SuppVideo1VelAccelprofiles.mp4
- SuppVideo2Gait.mp4
- SuppVideo38mazetask.mp4
- SuppVideo4Imaging.mp4
- SuppVideo5Ephys720p.mp4
